# Circulating soluble receptor for advanced glycation end products is decreased and inversely associated with acute phase response in chronic spontaneous urticaria

**DOI:** 10.1007/s00011-016-0914-5

**Published:** 2016-01-22

**Authors:** A. Kasperska-Zajac, A. Damasiewicz-Bodzek, K. Tyrpień-Golder, J. Zamlyński, A. Grzanka

**Affiliations:** Department of Internal Diseases, Dermatology and Allergology in Zabrze, SMDZ in Zabrze, Medical University of Silesia in Katowice, ul. M. Curie-Skłodowskiej 10, 41-800 Zabrze, Poland; Department of Chemistry in Zabrze, SMDZ in Zabrze, Medical University of Silesia in Katowice, Katowice, Poland; Department of Gynaecology, Obstetrics and Oncological Gynaecology, SMDZ in Zabrze, Medical University of Silesia in Katowice, Katowice, Poland

**Keywords:** Soluble receptor for advanced glycation end products, Chronic spontaneous urticaria, Acute phase response, C-reactive protein

## Abstract

**Background:**

Activation of receptor for advanced glycation end products (RAGE) leads to the proinflammatory response and the release of its soluble form (sRAGE) which appears to function as an anti-inflammatory feedback mechanism.

**Aim:**

To determine serum sRAGE concentration in CSU patients and its association with C-reactive protein (CRP) concentration, a nonspecific inflammatory marker of the disease activity.

**Methods:**

Concentrations of sRAGE and CRP were measured in serum of CSU patients and compared with the healthy controls.

**Results:**

Serum sRAGE concentrations were significantly decreased in CSU patients, especially those more severely affected. In addition, significant inverse correlations were observed between sRAGE and CRP concentrations.

**Conclusions:**

Down-regulation of sRAGE and its association with acute phase response suggest a role for RAGE activation in the pathogenesis of CSU. It seems that lower serum sRAGE concentration may enhance the urticarial processes.

## Introduction

The pathogenesis of chronic spontaneous urticaria (CSU) is complex and involves a number of mediators [1–4].

The receptor for advanced glycation end products (RAGE) is a member of the immunoglobulin superfamily of cell surface molecules, playing an important role in immune/inflammatory disorders [5–9]. This multiligand receptor binds a variety of molecules, including advanced glycation end products (AGEs), amyloid fibrils, S100/calgranulins and amphoterin, which lead to the inflammatory response and cellular dysfunction. It is able to increase vascular permeability and recruit leukocytes into sites of inflammation [9, 10]. The soluble form of RAGE (sRAGE) prevents the receptor-mediated signaling by neutralization/removal of the proinflammatory ligands, acting as a decoy receptor [7, 11].

Circulating sRAGE has been found to be decreased in chronic inflammatory diseases [12].

In our previous study, we observed lower AEGs concentration in serum of CSU patients, despite the enhanced systemic inflammatory response. Paradoxical decrease of serum AGEs concentrations is probably a reflection of lower concentration of the ‘negative acute phase proteins’, such as albumin [13]. It seems interesting to perform further investigations of the RAGE signaling system in the disease. Thus, the aim of this study was to determine serum sRAGE concentration in CSU patients and its association with C-reactive protein (CRP) concentration, a nonspecific inflammatory marker of the disease activity.

## Materials and methods

37 patients with active CSU (26 women and 11 men; mean age: 39 ± 9.5 years) with a median disease duration of 2.9 years were enrolled in the study, as described previously [13].

In all cases, any known causes of CSU were ruled in (out) by appropriate investigations. Each patient underwent the following tests: routine laboratory tests (FBC, urine analysis, ESR, C-reactive protein, serum glucose, hepatic functions, and creatinine), stool (for parasites), hepatitis serology, antinuclear and antithyroid microsomal antibodies, thyroid function tests, chest X-ray and abdominal ultrasonography. Additionally, dental, gynecological and ENT consultations as well as the autologous serum skin test (ASST) [14] were performed.

The urticaria activity score (UAS) was estimated during 4 days and on the blood sampling day to be graded as follows: mild (0–8), moderate (9–16) and severe (17–24). The study comprised 20 patients with mild and 17 patients with moderate-severe urticaria symptoms.

None of the examined subjects had taken any oral corticosteroids within 5 weeks or antihistamines within at least 5 days before the study.

The control group consisted of 24 healthy individuals (13 women and 11 men), of comparable age (41.3 ± 8.2 years) and BMI (<30).

The Ethics Committee of the Medical University of Silesia approved of the study and written, informed consent was obtained from all the subjects participating.

### RAGE concentration assay

sRAGE in tested serum samples were determined using Human RAGE/AGER ELISA Kit (Sigma-Aldrich, Saint Louis, MO) according to manufacturers’ recommendations. Absorbances were read with the Power Wave XS plate reader (BioTek, Winooski, VT) at 450 nm (reference wave length −630 nm), and results processed with the KC Junior software (BioTek, Winooski, VT).

Determinations were done during one series. The intra-assay variation was 8 %. The assay sensitivity was 3 pg/ml.

### Assay of CRP concentration

Serum C-reactive protein (CRP) concentrations were assayed using Roche/Hitachi cobas c system. Normal lab ranges: lower than 5.0 mg/l.

### Autologous serum skin test (ASST)

Intradermal ASST was performed following the method by Sabroe et al. [14]. Serum-induced a red wheal response of diameter greater by at least 1.5 mm than that of the control wheal, induced by physiological saline, was accepted as positive. Skin prick test with histamine served as a positive control.

### Statistical analysis

The obtained results were presented using basic parameters of descriptive statistics, such as median value, quartile range, mean value and standard deviation. Normal distribution of data was measured using Shapiro–Wilk’s test. Independent data between the groups of patients with CSU and the control group and between CSU patients with mild and moderate-severe symptoms were compared using non-parametric *U* Mann–Whitney test. The Spearman’s rank test was used for correlations. The values of *p* < 0.05 were considered statistically significant. Calculations were performed with STATISTICA for Windows 10.0 software (StatSoft, Cracow, Poland).

#### Serum sRAGE concentration

**Fig. 1:**
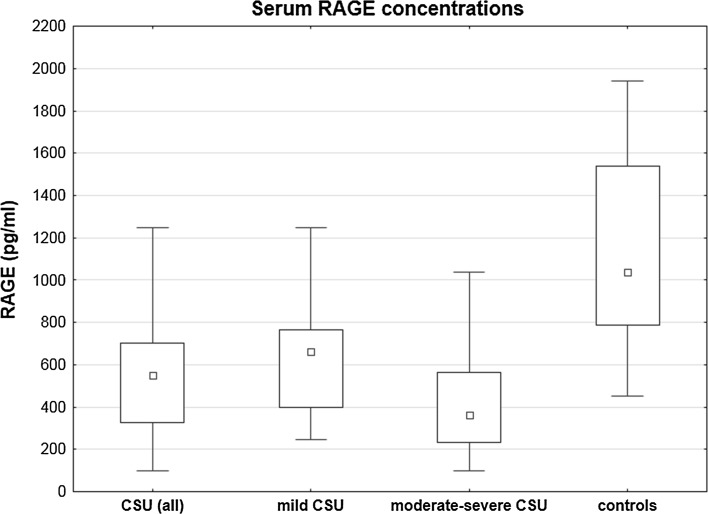
Serum RAGE concentration in the healthy subjects and chronic spontaneous urticaria (CSU) patients with different disease activity. CSU (all) versus controls, *p* < 0.00001; moderate-severe CSU versus mild CSU versus controls, *p* < 0.00001 and *p* < 0.01, respectively; mild CSU versus controls, *p* < 0.001

Serum concentration of sRAGE was significantly lower in CSU patients as compared with the healthy subjects (median 551.4 vs. 1037.25 pg/ml; *p* < 0.00001; Fig. 1).

sRAGE serum concentration was significantly lower in moderate-severe CSU patients as compared with those with mild CSU and the healthy subjects (median 362.99 vs. 663.85 vs. 1037.25 pg/ml, *p* < 0.00001 and *p* < 0.01, respectively). In addition, sRAGE concentration in mild CSU patients was significantly lower than in the health subject (663.85 vs. 1037.25 pg/ml, *p* < 0.001).

No significant differences in sRAGE concentrations between ASST(+) and ASST(−) CSU patients (selected according to the similar UAS) were observed.

#### Serum CRP concentration

Serum CRP concentrations were significantly higher in CSU patients as compared with the healthy subjects (median 2.85 vs. 1.02 mg/l, *p* < 0.00001). CRP concentration in moderate-severe CSU group was significantly higher than in mild CSU group and the healthy subjects (median 10.30 vs. 1.2 vs. 1.02 mg/l, *p* < 0.00001). There was no significant difference in serum CRP concentration between patients with mild CSU and the control group (median 1.2 vs. 1.02 mg/l, *p* = 0.07) [13].

#### Associations

There was a significant negative correlation between sRAGE and CRP concentrations in CSU patients (*R* = −0.35, *p* = 0.03), but not in the healthy subjects (*R* = 0.24, *p* = 0.26). There were no significant correlations between sRAGE and AGEs [13] concentrations, neither in CSU patients (*R* = −0.13, *p* = 0.44) nor in the healthy controls (*R* = 0.29, *p* = 0.12).

## Discussion

To the best of our knowledge, this study is the first to demonstrate any association between serum sRAGE concentrations and the urticarial processes.

We provide evidence that serum sRAGE concentration is decreased in CSU patients as compared with the healthy subjects, and it is inversely associated with CRP—a marker of CSU activity and the inflammatory response [2, 3]. These data suggest that such lowers sRAGE concentration in CSU patients could be considered as a reflection of their inflammatory status.

Nevertheless, the mechanism and clinical significance of decreased circulating sRAGE concentrations in CSU are currently unclear.

Theoretically, lower sRAGE concentrations in CSU patients can be explained by different mechanisms. It seems likely that similarly to other diseases, associated with the systemic inflammatory response, there is a wide diversity of RAGE ligands in the inflamed skin and/or in the circulation, that could bind and eliminate sRAGE, leading to lower circulating concentrations of sRAGE in CSU [10, 11]. Another intriguing possibility is that, either primary or secondary dysregulation of RAGE system leads to insufficient sRAGE production in CSU.

The role for circulating sRAGE in CSU is currently unknown.

It is known, that sRAGE regulates RAGE-mediated processes by contributing to the removal/neutralization of circulating ligands and appears to function as an anti-inflammatory feedback mechanism [11, 15]. In addition, it has been demonstrated that decreased sRAGE concentrations is associated with the increased inflammation in various disorders [12, 16]. On the other hand, higher plasma concentration of sRAGE reduces the risk of inflammatory complications in several chronic diseases [7, 11]. Consequently, one might speculate, that decreased concentration of sRAGE in CSU may increase the propensity towards inflammation by facilitating RAGE-dependent cellular activation and proinflammatory response, similarly to other diseases/conditions [5, 6, 9, 12].

So far, it has only been our previous study to focus on the involvement of the AGEs–RAGE axis in CSU. We found that CSU patients had significantly decreased serum AGEs concentrations, as compared with the healthy subjects [13]. However, serum sRAGE concentrations were found not to correlate with circulating AGEs concentrations, suggesting interactions with other proinflammatory ligands, including amyloid fibrils, S100/calgranulins and amphoterin.

In the present study, serum sRAGE concentration was significantly lower in moderate-severe CSU patients, as compared with those with mild CSU and the healthy subjects. In addition, sRAGE concentration in mild CSU patients was significantly lower than in the healthy subjects.

However, as serum sRAGE concentrations were not compared between moderate and severe CSU patients, and because of a relatively small study group, further research is necessary to address the issues of precise relation between circulating sRAGE and the disease activity/severity.

## Conclusions

Serum sRAGE concentrations were significantly decreased in CSU patients, especially those more severely affected, and were inversely related to CRP, a nonspecific inflammatory marker of the disease activity. Down-regulation of sRAGE and its association with the acute phase response suggest a role for RAGE activation in the pathogenesis of CSU. It seems that lower serum sRAGE concentration may enhance the urticarial processes.

## References

[1] Asero R, Cugno M, Tedeschi A (2011). Activation of blood coagulation in plasma from chronic urticaria patients with negative autologous plasma skin test. J Eur Acad Dermatol Venereol.

[2] Kasperska-Zajac A, Grzanka A, Damasiewicz-Bodzek A (2015). IL-6 signaling in chronic spontaneous urticaria. PLos One.

[3] Grzanka A, Machura E, Misiolek M, Mazur B, Jochem J, Kasperski J, Kasperska-Zajac A (2014). Relationship between vitamin D status and the inflammatory state in patients with chronic spontaneous urticaria. J Inflamm (Lond).

[4] Kasperska-Zajac A, Grzanka A, Misiolek M, Mazur B, Machura E (2015). Pentraxin-3 as a local inflammatory marker in chronic spontaneous urticaria. Cytokine.

[5] Hofmann MA, Drury S, Fu C, Qu W, Taguchi A, Lu Y, Avila C, Kambham N, Bierhaus A, Nawroth P, Neurath MF, Slattery T, Beach D, McClary J, Nagashima M, Morser J, Stern D, Schmidt AM (1999). RAGE mediates a novel proinflammatory axis: a central cell surface receptor for S100/calgranulin polypeptides. Cell.

[6] Ramasamy R, Yan SF, Schmidt AM (2009). RAGE: therapeutic target and biomarker of the inflammatory response—the evidence mounts. J Leukoc Biol.

[7] Geroldi D, Falcone C, Emanuele E (2006). Soluble receptor for advanced glycation end products: from disease marker to potential therapeutic target. Curr Med Chem.

[8] Neeper M, Schmidt AM, Brett J, Yan SD, Wang F, Pan YC, Elliston K, Stern D, Shaw A (1992). Cloning and expression of a cell surface receptor for advanced glycosylation end products of proteins. J Biol Chem.

[9] Chavakis T, Bierhaus A, Al-Fakhri N, Schneider D, Witte S, Linn T, Nagashima M, Morser J, Arnold B, Preissner KT, Nawroth PP (2003). The pattern recognition receptor (RAGE) is a counter receptor for leukocyte integrins: a novel pathway for inflammatory cell recruitment. J Exp Med.

[10] Stern D, Yan SD, Yan SF, Schmidt AM (2002). Receptor for advanced glycation endproducts: a multiligand receptor magnifying cell stress in diverse pathologic settings. Adv Drug Deliv Rev.

[11] Moser B, Hudson BI, Schmidt AM (2005). Soluble RAGE: a hot new biomarker for the hot joint?. Arthritis Res Ther.

[12] Pullerits R, Bokarewa M, Dahlberg L, Tarkowski A (2005). Decreased levels of soluble receptor for advanced glycation end products in patients with rheumatoid arthritis indicating deficient inflammatory control. Arthritis Res Ther.

[13] Grzanka A, Damasiewicz-Bodzek A, Machura E, Szumska M, Tyrpień-Golder K, Mazur B, Kasperska-Zajac A (2014). Chronic spontaneous urticaria is characterized by lower serum advanced glycation end-products. Biomed Res Int.

[14] Sabroe RA, Grattan CEH, Francis DM, Barr RM, Kobza Black A, Greaves MW (1999). The autologous serum skin test: a screening test for autoantibodies in chronic idiopathic urticatia. Br J Dermatol.

[15] Pullerits R, Brisslert M, Jonsson IM, Tarkowski A (2006). Soluble receptor for advanced glycation end products triggers a proinflammatory cytokine cascade via β2 integrin Mac-1. Arthritis Rheum.

[16] Falcone C, Emanuele E, D’Angelo A, Buzzi MP, Belvito C, Cuccia M, Geroldi D (2005). Plasma levels of soluble receptor for advanced glycation end products and coronary artery disease in nondiabetic men. Arterioscler Thromb Vasc Biol.

